# Effects of Socioeconomic Status and Social Support on Violence against Pregnant Women: A Structural Equation Modeling Analysis

**DOI:** 10.1371/journal.pone.0170469

**Published:** 2017-01-20

**Authors:** Marizélia Rodrigues Costa Ribeiro, Antônio Augusto Moura da Silva, Maria Teresa Seabra Soares de Britto e Alves, Rosângela Fernandes Lucena Batista, Cecília Cláudia Costa Ribeiro, Lilia Blima Schraiber, Heloisa Bettiol, Marco Antônio Barbieri

**Affiliations:** 1 Department of Medicine III, Federal University of Maranhao, Sao Luis, Maranhao, Brazil; 2 Department of Public Health, Federal University of Maranhao, Sao Luis, Maranhao, Brazil; 3 Department of Odontology, Federal University of Maranhao, Sao Luis, Maranhao, Brazil; 4 Department of Preventive Medicine, Faculty of Medicine, University of Sao Paulo, Sao Paulo, Sao Paulo, Brazil; 5 Department of Pediatrics, Faculty of Medicine of Ribeirao Preto, University of Sao Paulo, Ribeirao Preto, Sao Paulo, Brazil; Medizinische Universitat Wien, AUSTRIA

## Abstract

Few studies have used structural equation modeling to analyze the effects of variables on violence against women. The present study analyzed the effects of socioeconomic status and social support on violence against pregnant women who used prenatal services. This was a cross-sectional study based on data from the Brazilian Ribeirão Preto and São Luís birth cohort studies (BRISA). The sample of the municipality of São Luís (Maranhão/Brazil) consisted of 1,446 pregnant women interviewed in 2010 and 2011. In the proposed model, socioeconomic status was the most distal predictor, followed by social support that determined general violence, psychological violence or physical/sexual violence, which were analyzed as latent variables. Violence was measured by the World Health Organization Violence against Women (WHO VAW) instrument. The São Luis model was estimated using structural equation modeling and validated with 1,378 pregnant women from Ribeirão Preto (São Paulo/Brazil). The proposed model showed good fit for general, psychological and physical/sexual violence for the São Luís sample. Socioeconomic status had no effect on general or psychological violence (p>0.05), but pregnant women with lower socioeconomic status reported more episodes of physical/sexual violence (standardized coefficient, SC = -0.136; p = 0.021). This effect of socioeconomic status was indirect and mediated by low social support (SC = -0.075; p<0.001). Low social support was associated with more episodes of general, psychological and physical/sexual violence (p<0.001). General and psychological violence indistinctly affected pregnant women of different socioeconomic status. Physical/sexual violence was more common for pregnant women with lower socioeconomic status and lower social support. Better social support contributed to reduction of all types of violence. Results were nearly the same for the validation sample of Ribeirão Preto except that SES was not associated with physical/sexual violence.

## Introduction

Violence against pregnant women seems to be a more frequent situation than the obstetrical complications commonly investigated during prenatal care, such as gestational diabetes [1]. Its rates range from 0.9% to 57.1% depending on the definitions and types of violence investigated, the methodologies used and on sociocultural differences [1–6]. In Brazil, the WHO Multi-Country Study On Women’s Health and Domestic Violence Against Women detected an 8% prevalence of abuse of pregnant women in São Paulo and an 11.1% prevalence in the Forest Zone of Pernambuco [7].

Because of these high rates and negative repercussions on the health and life of women and their children, several studies have sought to identify factors associated with violence during pregnancy, among them, socioeconomic status (SES) and social support [1,2,4,8].

SES was defined as the position that the individual or group occupies in a society. It is a multidimensional construct most commonly measured as a combination of education, occupation and income [9,10]. In a literature review of studies conducted in countries on different continents, the association between SES and violence against pregnant women was considered to be inconclusive either because the variables representing SES were determined without taking into account measurement error, or because the analyses were not adjusted, or because of socioeconomic homogeneity of the samples [1]. However, a review of African studies revealed low SES as a risk factor for intimate partner violence [2]. In Brazil, two studies investigating the association between socioeconomic characteristics of pregnant women and intimate partner violence showed contrasting results [11,12]. In the WHO Multi-Country Study, high SES was a protective factor for intimate partner violence [13], but this finding was not replicated in the sample collected in São Paulo (Brazil) [14].

Less investigated than SES [1,2,5,8], social support concerns the different resources offered by a social network to persons in situations of need [15,16]. Two studies analyzed in a literature review showed that social support was a protective factor against physical violence, although a third one did not detect this association [1]. These studies measured social support in different manners and used multiple logistic regression in the analyses [17–19].

Most studies investigated physical or sexual violence and a few studied psychological violence against pregnant women [1,20,21]. Recently a multidimensional construct of general violence composed of physical, sexual and psychological components based on the WHO Violence Against Women questionnaire has been validated [22] but there are no studies that used this definition available to date.

Given the inconsistencies across studies to date, the present research aimed to answer the following questions. How do general violence vary according to SES? Is the effect of SES similar for psychological and physical/sexual violence? Does social support mediate the association between SES and violence? The influences of SES and social support on violence against pregnant women were first examined in prenatal care services in the municipality of São Luís (Maranhão/Brazil) and replicated with data obtained in the municipality of Ribeirão Preto (São Paulo/Brazil).

## Methods

This cross-sectional study used data from the Brazilian Ribeirão Preto and São Luís birth cohort studies (BRISA), which investigated the etiological factors of preterm birth in the municipalities of São Luís (Maranhão/Brazil) and Ribeirão Preto (São Paulo/Brazil) in 2010 and 2011. In both municipalities, data were collected in a similar way.

### The municipality of São Luís

The municipality of São Luís, capital city of the state of Maranhão, is located in the Northeast region of Brazil. In 2010, its population was 1,014,837 inhabitants, with a mean per capita monthly family income of R$ 805.36 (approximately US$ 446.00). The percentage of the economically active population that was unemployed was 11.96% [23]. In 2011, only 41.4% of the women giving birth to liveborn infants attended seven or more prenatal visits [24].

### The municipality of Ribeirão Preto

The municipality of Ribeirão Preto is located in the state of São Paulo, Southeast region of Brazil. In 2010, its population was 604,682 inhabitants, the mean per capita monthly family income was R$ 1,314.04 (approximately US$ 728.00) and the unemployment rate was 4.72% [25]. In 2011, 77.3% of all pregnant women attended at least seven prenatal care visits in this city [26].

### Participants and samples

Pregnant women users of public and private prenatal services and who wanted to give birth in the municipality where data collection took place were invited to participate in the study if the following criteria were met: a) gestational age of less than 22 weeks; b) obstetric ultrasound performed with less than 20 weeks of gestational age; and c) singleton pregnancy. Data collection was performed from the 22^nd^ to the 25^th^ week of gestational age.

Convenience samples were studied in the two municipalities due to the difficulty of obtaining a random sample from the population of pregnant women. From February 2010 to June 2011, 1,447 pregnant women participated in the BRISA cohort in São Luís. One pregnant woman was excluded from the study because she did not respond to the questions about violence. The final Ribeirão Preto sample consisted of 1,378 pregnant women whose data were collected from February 2010 to February 2011.

The present investigation was approved by the Research Ethics Committees of the University Hospital of the Federal University of Maranhão (protocol nº 4771/2008-30) and of the University Hospital of the Faculty of Medicine of Ribeirão Preto (protocol nº 4116/2008). The investigators declare that they have no conflicts of interest.

All women gave written informed consent to participate in the study and for those younger than 18 an accompanying adult also signed the consent form. All subjects were informed that the BRISA prenatal cohort was investigating risk factors for preterm birth, and that confidentiality, image protection and non-stigmatization were guaranteed to all participants.

### Collection and storage of BRISA data

Two questionnaires were used for data collection: the Self-Applied Prenatal Questionnaire and the Prenatal Interview Questionnaire. The questions about violence and social support were part of the self-applied questionnaire. Education of the pregnant woman, occupation of the family head, monthly family income and economic class were collected by the interviewers and composed the Prenatal Interview Questionnaire.

### Theoretical model and variables

**Fig 1:**
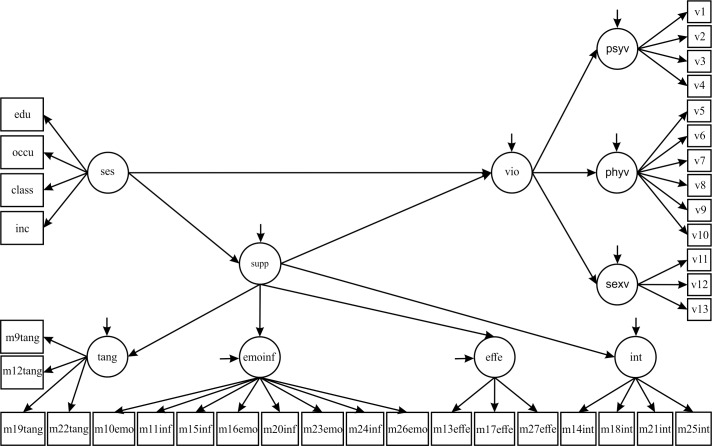
Structural equation modeling of general violence against pregnant women in São Luís, 2010–2011. ses: socioeconomic status. class: economic class. inc: family income. occu: occupation of the family head. edu: years of study of the pregnant woman. vio: general violence was analyzed as a three-dimensional second-order factor structure. Questions about psychological (v1-v4), physical (v5-v10) and sexual violence (v11-v13) were presented in the text that described the validation of the WHO VAW (World Health Organization–Violence against Women) instrument for this population of pregnant women [22]. psyv: psychological violence. phyv: physical violence. sexv: sexual violence. supp: social support. int: positive social interaction support (m14int, m18int, m21int, m25int). emoinf: emotional/informational support (m10emo, m11inf, m15inf, m16emo, m20inf, m23emo, m24inf, m26emo). affe: affectionate support (m13affe, m17affe, m27affe). tang: tangible support (m9tang, m12tang, m19tang, m22tang).

In the initially proposed theoretical model (Fig 1), socioeconomic status (SES) occupied the most distal position, determining social support (supp) that led to general violence (vio), psychological violence (psyv) or physical/sexual (physexv) violence.

The following latent variables (or constructs) were used: socioeconomic status, social support, general violence, psychological violence and physical/sexual violence.

Socioeconomic status was based on four indicators: a) education, measured as years of study of the pregnant woman (edu), categorized as up to 4 years, 5 to 8 years, 9 to 11 years, and 12 years or more; b) occupation of the family head (occu), categorized as unskilled manual, semi-specialized manual, office duties, high-level professional, and administrator/manager/director/owner; c) monthly family income in minimum wages (inc), categorized as less than 1 national minimum wage (approximately US$ 290.00) for 2010, 1 to less than 3, 3 to less than 5, and 5 or more; and d) economic class (class), categorized as D/E, C and A/B, consisting of ownership of assets and educational level of the family head, with categories A and B having greater purchasing power.

The instrument from the Brazilian Association of Research Companies was used to measure economic class, based on the family head’s educational level and consumer goods ownership (color television, radio, bathroom, automobile, full-time maid, washing machine, videocassette and/or DVD player, freezer, and refrigerator) [27].

Social support (supp) was elaborated based on the following four dimensions, proposed by the Medical Outcomes Study (MOS): tangible support (four questions), emotional/informational support (seven questions), affectionate support (three questions), and positive social interaction support (four questions) [15].

The 13 questions measuring violence were obtained from the Brazilian version of the World Health Organization Violence against Women (WHO VAW) instrument. The first four were related to psychological (emotional) violence and asked about insults, humiliations and threats. Six questions investigated physical violence and asked about slaps, threats or wounds with objects, shoves, jerking/shaking, punches, kicks, beatings, strangling, purposeful burns, threat or wound with a firearm, knife or other type of weapon. The three questions about sexual violence asked about forced sexual relations. The response options for each of these questions were none, once, a few times, and many times [28]. Types of abuse on the part of different perpetrators were counted, both in the domestic-family environment and in other spaces, including abuse by unknown persons.

The instruments used to investigate social support [29] and violence [22,30] were previously validated in Brazil.

### Descriptive analysis and structural equation modeling

In the descriptive analysis, violence was considered to have occurred when the interviewed woman responded affirmatively to at least one of the 13 questions. Frequencies, percentages and the p-value of the Chi-squared test were calculated using Stata software, version 12.0 (College Station, Texas, USA).

Structural equation modeling (SEM), a statistical method that performs confirmatory factor analysis and simultaneously estimates a series of multiple regression equations assessing the direct and indirect effects of the variables on the outcome, was used [31–34]. Since all variables were declared as categorical, the mean-and-variance-adjusted weighted least squares estimator with theta parameterization was used. This step was carried out using Mplus software, version 7.31 (Los Angeles, California, USA).

In the initial proposed model (Fig 1), SES was investigated as a first-order factor and social support as a second-order factor composed of four dimensions: tangible, emotional/informational, affectionate and positive social interaction support. General violence was analyzed as a three dimensional second-order factor structure, consisting of psychological, physical and sexual dimensions. We also investigated the effects of SES and social support on psychological violence (first-order factor) and physical/sexual violence (two-dimensional second-order factor).

We hypothesized violence as a reflective model because: a) the indicators used to measure violence were related conceptually because they are thought to have a common cause; and b) the indicators that measure violence were correlated (high internal consistency) [31].

Multiple-sample analyses of measurement invariance was performed for the three ways of operationalizing violence: 1) violence as a single three-dimensional second-order latent variable assessing generalized violence, composed of three domains (psychological, physical and sexual); 2) a first-order latent variable for psychological violence; and 3) a second-order latent variable consisting of physical and sexual sub-domains. Initially baseline models based on the proposed theoretical structure were performed for each city. Then a configural model with the same factor structure was tested, with no equality constraints imposed. Finally, we tested for weak invariance (invariant factor loadings). The chi-square difference test was used to compare the two nested models (the one with imposing invariant factor loadings with the configural model). If p>0.05 the two models were considered equivalent and the hypothesis of invariant factor loadings was not rejected [32].

To determine whether the models showed good fit we considered: a) p-value (p) of more than 0.05 for the Chi-square test (χ^2^) [31]; b) Root Mean Square Error of Approximation (RMSEA) values are close to 0.06 or below [33]; c) values close or higher than 0.95 for the Comparative Fit Index and the Tucker Lewis Index (CFI/TLI) [33]; and d) Weighted Root Mean Square Residual (WRMR) values of 1 or lower [34], still an experimental fit index [32].

In the analyses of the standardized estimates for the construction of the latent variables, a factor loading higher than 0.5 with p<0.05 was considered to indicate that the correlation between the indicator variable and the construct was of moderately high magnitude [31].

The **modindices** command was used for suggestions of modifications of the initial hypothesis. When the proposed modifications were considered to be plausible from a theoretical viewpoint, a new model was elaborated and analyzed if the value of the modification index were higher than 10 [34].

The direct and indirect effects of the latent and observed variables were assessed in the final model elaborated for general violence, with an effect being considered to be present when p<0.05. This final model was also used for the analyses of psychological and physical/sexual violence separately.

The final São Luís models were tested using the sample of the municipality of Ribeirão Preto (São Paulo/Brazil) (N = 1,378) for validation.

## Results

The characteristics of the sample in São Luís are presented in Table 1. Of the interviewed women, 75% had completed 9 to 11 years of education. The percentages of pregnant women in families of economic class C and receiving less than one minimum wage were 68% and 5%, respectively. About 75% of the family heads had manual occupations.

**Table 1:** Characteristics of pregnant women, intimate partners and family heads in São Luís-Brazil, 2010–2011.

Variables	Total		General violence^a^		
	N	%	n	%	p^b^
**Economic class: Brazil**^**c**^	1,379				0.019
** D/E**	225	16.3	124	55.1	
** C**	933	67.7	449	48.2	
** A/B**	221	16.0	109	49.8	
**Family income (in minimum wages)**	1,403				0.005
** Less than 1**	70	5.0	40	58.0	
** 1 to less than 3**	787	56.1	370	47.0	
** 3 to less than 5**	333	23.7	168	50.8	
** 5 or more**	213	15.2	117	54.9	
**Occupation of the family head**	1,364				0.529
** Unskilled manual laborer**	396	29.0	202	51.0	
** Semi-specialized manual laborer**	564	41.4	262	46.7	
** Specialized manual laborer**	66	4.8	30	45.4	
** Office duties**	218	16.0	110	50.5	
** High-level professional**	77	5.7	43	56.6	
** Administrator/manager/director/owner**	43	3.1	21	48.8	
**Years of study of the pregnant woman**	1,445				0.332
** 0 to 4**	21	1.15	11	52.4	
** 5 to 8**	162	11.2	81	50.3	
** 9 to 11**	1,090	75.4	529	48.7	
** 12 or more**	172	11.9	94	54.6	

^a^ General violence was categorized as a dichotomous variable (yes and no) using the STATA software, version 12.0. Questions about psychological, physical and sexual violence were presented in the text that described the validation of the WHO VAW (World Health Organization–Violence against women) instrument for this population of pregnant women [22].

^b^ p-value obtained by the chi-squared test.

^c^ economic class was categorized as D/E, C and A/B, consisting of ownership of assets and educational level of the family head, with categories A and B having greater purchasing power.

**Fig 2:**
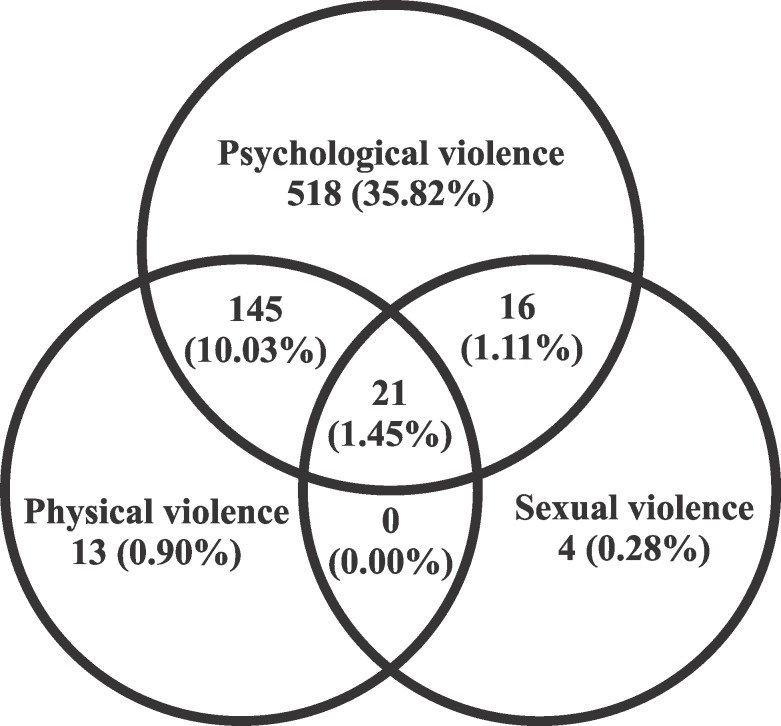
Venn diagram showing frequencies and percentages of psychological, physical and sexual violence against pregnant women in São Luís, 2010–2011.

The rate of general violence was 49.6%. The percentages of psychological, physical and sexual violence were 48.4%, 12.4% and 2.8%, respectively (Fig 2). For the validation sample of Ribeirão Preto, the rate of general violence was 43.6%, of psychological violence 42.9%, of physical violence 10.5% and of sexual violence 2.1%.

Intimate partners were the main perpetrators of violence, and were responsible for 47.5% of all reports. Family members accounted for 31.3% and non-family members were cited in 21.2% of cases. The percentage of physical intimate partner violence was 66%.

In the confirmatory factor analysis all hypothesized models showed good fit for São Luís and Ribeirão Preto (Table 2). Some suggested modification indices for the measurement models were >10 but were not considered plausible. Tests for weak invariance (invariant factor loadings) were not significant.

**Table 2:** Multiple-sample analysis of measurement invariance of the WHO VAW model for São Luis and Ribeirão Preto: indices of model fit and chi-square difference-test statistics. Brazil, 2010–2011.

Models	χ ^2^ valor ^f^	χ ^2^^g^ DF	RMSEA^h^	CFI^i^	TLI^j^	WRMR^k^	Model^l^ Comparison	Difference Testing^m^	DF Difference^n^	p^o^
**General violence**^a^										
**1. São Luís**	134.413	62	0.028	0.986	0.982	0.962				
**2. Ribeirão Preto**	163.863	62	0.035	0.989	0.987	0.992				
**3.Configural model**^d^	330.598	150	0.029	0.988	0.988	1.446				
**4. Invariant factor loadings**^**e**^	312.192	160	0.026	0.990	0.990	1.487	Model 4 versus 3	9.550	10	0.481
**Psychological violence**^**b**^										
**5. São Luís**	29.078	2	0.097	0.990	0.971	0.942				
**6. Ribeirão Preto**	25.940	2	0.093	0.993	0.979	0.798				
**7. Configural model**^**d**^	66.433	12	0.057	0.991	0.991	1.447				
**8. Invariant factor loadings**^**e**^	59.478	15	0.046	0.993	0.994	1.507	Model 8 versus 7	3.125	3	0.372
**Physical/sexual violence**^**c**^										
**9. São Luís**	45.923	26	0.023	0.992	0.989	0.716				
**10. Ribeirão Preto**	63.987	26	0.033	0.994	0.992	0.768				
**11. Configural model**^**d**^	126.183	70	0.024	0.994	0.994	1.138				
**12. Invariant factor loadings**^**e**^	119.636	77	0.020	0.996	0.996	1.187	Model 12 versus 11	5.712	7	0.574

^a^ General violence was analyzed as a three dimensional second-order factor structure. Questions about these three types of violence were presented in the text that described the validation of the WHO VAW (World Health Organization–Violence against Women) instrument for this population of pregnant women [22].

^b^ Psychological violence was analyzed as a first-order factor structure.

^c^ Physical/sexual violence was analyzed as a two-dimensional second-order factor structure, including physical and sexual violence.

^d^ Model with no equality constraints.

^e^ Model with all factor loadings invariant (weak invariance).

^f^ Chi-squared test.

^g^ Degrees of freedom.

^h^ Root Mean Square Error of Approximation.

^i^ Comparative Fit Index.

^j^ Tucker Lewis Index.

^k^ Weighted Root Mean Square Residual.

^l^ Comparison between models.

^m^ Chi square for difference testing (difftest command in MPlus).

^n^ Difference in the models’ degrees of freedom.

^o^ p-value for the chi square for difference testing.

The proposed structural model (Fig 1) showed good fit for the São Luís sample and there were no plausible suggestions of modification considering general, psychological and physical/sexual violence. The final models for general violence, psychological violence and physical/sexual violence were tested using the BRISA prenatal cohort sample of Ribeirão Preto and RMSEA, CFI and TLI indicators also showed good fit (Table 3).

**Table 3:** Fit indices of models for general, psychological and physical/sexual violence against pregnant women in São Luís and Ribeirão Preto-Brazil, 2010–2011.

Models		χ ^2^^e^	RMSEA^g^			CFI^i^	TLI^j^	WRMR^k^
		Valor	DF^f^	p-value	valor				90%CI^h^	p-value
**São Luís**										
**General violence**^a^	1.208.013		584	<0.001	0.027	0.025–0.029	0.999	0.990	0.989	1.352
**Psychological violence**^b^	1.261.549		317	<0.001	0.045	0.043–0.048	0.998	0.985	0.984	1.530
**Physical/sexual violence**^c^	1.069.319		455	<0.001	0.031	0.028–0.033	0.999	0.990	0.989	1.376
**Ribeirão Preto**^**d**^										
**General violence**^a^	1.438.993		584	<0.001	0.032	0.030–0.034	0.999	0.989	0.989	1.583
**Psychological violence**^b^	1.760.082		317	<0.001	0.057	0.054–0.060	0.999	0.983	0.981	1.896
**Physical/sexual violence**^**c**^	1.408.589		455	<0.001	0.039	0.036–0.041	0.999	0.988	0.987	1.637

^a^ General violence was analyzed as a three dimensional second-order factor structure. Questions about these three types of violence were presented in the text that described the validation of the WHO VAW (World Health Organization–Violence against Women) instrument for this population of pregnant women [22].

^b^ Psychological violence was analyzed as a first-order factor structure.

^c^ Physical/sexual violence was analyzed as a two-dimensional second-order factor structure, including physical and sexual violence.

^d^ Results of the validation of the São Luís final model for the Ribeirão Preto sample.

^e^ Chi-squared test.

^f^ Degrees of freedom.

^g^ Root Mean Square Error of Approximation.

^h^ Confidence Interval.

^i^ Comparative Fit Index.

^j^ Tucker Lewis Index.

^k^ Weighted Root Mean Square Residual.

Each indicator of the latent variables had factor loadings higher than 0.5 with p-values of less than 0.001, except education for São Luís and occupation of the family head for Ribeirão Preto (Tables 4, 5 and 6).

**Table 4:** Standardized estimates, standard errors and p-values of direct and indirect effects of indicator variables and constructs on general violence against pregnant women in São Luís and Ribeirão Preto-Brazil, 2010/2011.

Paths	São Luís			Ribeirão Preto		
	Standardized estimate	Standard error	p-value	Standardized estimate	Standard error	p-value
Latent variables						
**ses**^**a**^						
** ses by**^**b**^ **class**^c^	0.801	0.041	<0.001	0.791	0.049	<0.001
** ses by inc**^**d**^	0.726	0.039	<0.001	0.647	0.040	<0.001
** ses by occu**^e^	0.558	0.038	<0.001	0.484	0.041	<0.001
** ses by edu**^f^	0.483	0.045	<0.001	0.629	0.047	<0.001
**vio**^**g**^						
** vio by phyv**^h^	0.863	0.060	<0.001	0.956	0.053	<0.001
** vio by psyv**^i^	0.856	0.057	<0.001	0.859	0.053	<0.001
** vio by sexv**^j^	0.727	0.070	<0.001	0.814	0.064	<0.001
**supp**^**k**^						
** supp by int**^l^	0.976	0.005	<0.001	0.988	0.004	<0.001
** supp by emoinf**^m^	0.944	0.005	<0.001	0.956	0.004	<0.001
** supp by affe**^n^	0.957	0.007	<0.001	0.948	0.006	<0.001
** supp by tang**^o^	0.847	0.012	<0.001	0.911	0.007	<0.001
**Direct effects**						
** vio on**^**p**^ **ses**	-0.015	0.045	0.734	-0.005	0.042	0.913
** vio on sup**	-0.281	0.043	<0.001	-0.288	0.039	<0.001
** supp on ses**	0.229	0.032	<0.001	0.189	0.033	<0.001
**Indirect effects**						
** ses to vio**						
** Total**	-0.080	0.042	0.061	-0.059	0.045	0.187
** Indirect**	-0.064	0.013	<0.001	-0.055	0.012	<0.001

^a^ ses: socioeconomic status.

^b^ by: MPUS command to derive latent variables.

^c ^class: economic class.

^d^ inc: family income.

^e^ occu: occupation of the family head.

^f ^edu: years of study of the pregnant woman.

^g^ vio: general violence was analyzed as a three dimensional second-order factor structure based on psychological, physical and sexual violence. Questions about these three types of violence were presented in the text that described the validation of the WHO VAW (World Health Organization–Violence against Women) instrument for this population of pregnant women [22].

^h^ phyv: physical violence.

^i^ psyv: psychological violence.

^j^ sexv: sexual violence.

^k^ supp: social support.

^l^ int: positive social interaction support.

^m^ emoinf: emotional/informational support.

^n^ affe: affectionate support.

^o^ tang: tangible support.

^p^ on: MPLUS command to estimate path coefficients.

**Table 5:** Paths, standard estimates, standard errors and p-values of direct and indirect effects of the observed variables and constructs regarding psychological violence against pregnant women in São Luís and Ribeirão Preto-Brazil, 2010–2011.

Paths	São Luís			Ribeirão Preto		
	Standardized estimate	Standard error	p-value	Standardized estimate	Standard error	p-value
Latent variables						
**ses**^**a**^						
** ses by**^**b**^ **class**^c^	0.800	0.040	<0.001	0.786	0.048	<0.001
** ses by inc**^d^	0.723	0.038	<0.001	0.644	0.040	<0.001
** ses by occu**^e^	0.564	0.037	<0.001	0.488	0.041	<0.001
** ses by edu**^f^	0.483	0.044	<0.001	0.635	0.047	<0.001
**psyv**^**g**^						
** v1**	0.830	0.026	<0.001	0.835	0.023	<0.001
** v2**	0.860	0.025	<0.001	0.907	0.023	<0.001
** v3**	0.752	0.029	<0.001	0.810	0.027	<0.001
** v4**	0.784	0.035	<0.001	0.826	0.035	<0.001
**supp**^**h**^						
** supp by int**^i^	0.976	0.005	<0.001	0.986	0.004	<0.001
** supp by emoinf**^j^	0.946	0.005	<0.001	0.958	0.004	<0.001
** supp by affe**^k^	0.956	0.007	<0.001	0.947	0.006	<0.001
** supp by tang**^l^	0.846	0.012	<0.001	0.910	0.007	<0.001
**Direct effects**						
** psyv on**^**m**^ **ses**	0.026	0.041	0.528	0.021	0.039	0.596
** psyv on supp**	-0.243	0.037	<0.001	-0.254	0.033	<0.001
** supp on ses**	0.229	0.032	<0.001	0.189	0.033	<0.001
**Indirect effects**						
** ses to psyv**						
** Total**	-0.030	0.040	0.457	-0,027	0.039	0.485
** Indirect**	-0.056	0.011	<0.001	-0.048	0.011	<0.001

^a^ ses: socioeconomic status.

^b^ by: MPUS command to derived latent variables.

^c ^class: economic class.

^d^ inc: family income.

^e^ occu: occupation of the family head.

^f ^edu: years of study of the pregnant woman.

^g^ psyv: psychological violence was analyzed as a first-order factor structure. Questions about psychological violence (v1-v4) were presented in the text that described the validation of the WHO VAW (World Health Organization–Violence against Women) instrument for this population of pregnant women [22].

^h^ supp: social support.

^i^ int: positive social interaction support.

^j^ emoinf: emotional/informational support.

^k^ affe: affectionate support.

^l^ tang: tangible support.

^m^ on: MPLUS command to estimate path coefficients.

**Table 6:** Paths, standard estimates, standard errors and p-values of direct and indirect effects of the observed variables and constructs regarding physical/sexual violence against pregnant women in São Luís and Ribeirão Preto-Brazil, 2010–2011.

	São Luís			Ribeirão Preto		
Paths	Standardized estimate	Standard error	p-value	Standardized estimate	Standard error	p-value
Latent variables						
**ses**^**a**^						
** ses by**^**b**^ **class**^c^	0.800	0.040	<0.001	0.788	0.049	<0.001
** ses by inc**^d^	0.727	0.039	<0.001	0.646	0.040	<0.001
** ses by occu**^e^	0.557	0.037	<0.001	0.487	0.041	<0.001
** ses by edu**^f^	0.486	0.045	<0.001	0.631	0.047	<0.001
**phys**^**g**^						
** v5**	0.936	0.028	<0.001	0.940	0.015	<0.001
** v6**	0.820	0.036	<0.001	0.920	0.016	<0.001
** v7**	0.910	0.030	<0.001	0.936	0.018	<0.001
** v8**	0.908	0.027	<0.001	0.991	0.012	<0.001
** v9**	0.849	0.073	<0.001	0.875	0.041	<0.001
** v10**	0.722	0.063	<0.001	0.886	0.044	<0.001
**sexv**^**h**^						
** v11**	0.945	0.045	<0.001	0.918	0.027	<0.001
** v12**	0.936	0.044	<0.001	0.977	0.028	<0.001
** v13**	0.889	0.062	<0.001	0.952	0.027	<0.001
**physexv**^**i**^						
** Phyv**	0.681	0.094	<0.001	0.859	0.111	<0.001
** Sexv**	0.842	0.118	<0.001	0.914	0.121	<0.001
**supp**^**j**^						
** supp by int**^k^	0.976	0.005	<0.001	0.986	0.004	<0.001
** supp by emoinf**^l^	0.946	0.005	<0.001	0.958	0.004	<0.001
** supp by affe**^m^	0.955	0.007	<0.001	0.946	0.006	<0.001
** supp by tang**^n^	0.847	0.012	<0.001	0.911	0.007	<0.001
**Direct effects**						
** physexv on**^o^ **ses**	-0.061	0.063	0.333	-0.035	0.057	0.537
** physexv on supp**	-0.328	0.060	<0.001	-0.300	0.057	<0.001
** supp on ses**	0.229	0.032	<0.001	0.189	0.033	<0.001
**Indirect effects**						
** ses to physexv**						
** Total**	-0.136	0.059	0.021	-0.092	0.062	0.138
** Indirect**	-0.075	0.017	<0.001	-0.057	0.015	<0.001

^a^ ses: socioeconomic status.

^b^ by: MPUS command to derive latent variables.

^c ^class: economic class.

^d^ inc: family income.

^e^ occu: occupation of the family head.

^f ^edu: years of study of the pregnant woman.

^g^ phyv: physical violence was analyzed as a two-dimensional first-order factorial structure. Questions about physical (v5-v10) and sexual violence (v11-v13) were presented in the text that described the validation of the WHO VAW (World Health Organization–Violence against Women) instrument for this population of pregnant women [22].

^h^ sexv: sexual violence.

^i^ physexv: physical/sexual violence.

^j^ supp: social support.

^k^ int: positive social interaction support.

^l^ emoinf: emotional/informational support.

^m^ affe: affectionate support.

^n^ tang: tangible support.

^o^ on: MPLUS command to estimate path coefficients.

SES had no effect on general or psychological violence (p>0.05) (Tables 4 and 5) in the BRISA prenatal cohort sample of São Luís, but women with low SES reported more physical/sexual violence (standardized coefficient, SC = -0.136; p = 0.021). Women of low SES experienced more physical/sexual violence only indirectly (SC = -0.075 and p = 0.017), via low social support (Table 6).

For the São Luís sample, the higher the social support the lower the reports of general, psychological and physical/sexual violence. Furthermore, for these three outcomes, the higher the SES the higher the social support (Tables 4, 5 and 6).

The same associations for SES and social support were obtained for the validation sample of Ribeirão Preto for general violence and psychological violence (Tables 4 and 5). However, SES was not associated with physical/sexual violence, although social support still had a protective effect for this type of violence against women.

## Discussion

In the BRISA prenatal cohort, psychometric analysis of the latent variables used to measure general, psychological and physical-sexual violence showed the same factor structure and invariant factor loadings in the São Luís and Ribeirão Preto samples.

In the municipality of São Luís, general and psychological violence against pregnant women occurred at similar rates in all socioeconomic levels. The direct/positive and indirect/negative effects canceled out the total effect of SES on these two outcomes. However, pregnant women with low SES suffered more physical/sexual violence, an effect that was fully indirect/negative and mediated by low social support.

The effects of SES on general violence were similar to those for psychological violence, which was four times more frequent than physical violence, the second most prevalent type, and 16 times more frequent than sexual violence. For this same population of pregnant women, the use of a Poisson regression model with a hierarchical approach at three levels also did not detect an association between variables measuring socio-economic status (schooling, remunerated job, family income or economic class of the pregnant woman) and exclusive psychological violence or recurrent exclusive psychological violence [20].

Physical/sexual violence predominated in the lowest socioeconomic strata in the BRISA prenatal cohort sample of São Luís. The total effect of SES occurred indirectly, mediated by low social support. One possible explanation for this finding is that high social support decreases the negative effects of poverty on violence, thus reducing the vulnerability of low SES mothers to violence [35].

In the BRISA prenatal cohort sample of São Luís intimate partners were the main perpetrators of physical violence. It is possible to explain this effect of socioeconomic status from male domination standards. From this perspective, social and structural contexts contribute to shaping values and norms also in the domestic-family environment, including gender relations. Intimate partners from families with lower socioeconomic status experience high levels of stress because they have not achieved financial success, and may try to strengthen their dominance, showing strength and power through violence [36,37].

In the validation model of physical/sexual violence for the Ribeirão Preto sample, SES was not associated with this type of violence. This may be due to the higher socioeconomic level of this municipality compared to the São Luís sample.

Low socioeconomic levels have been frequently found to be associated with violence. However, the investigators used one or more variables representing this condition and their conclusions were based on bivariate or multivariable analyses. No previous study has used structural equation modeling, which allows the examination of mediating pathways [1,4]. Violence by intimate partners has been suggested to be related to gender inequality more than to SES [38].

Possible explanations for the divergence between the present results and those reported by others regarding SES could be the sociocultural diversity across studies, the different types of violence investigated, the variations of the period of women’s life when the data were collected (pregnancy, puerperium or other periods) and methodological characteristics (selection of sample, of instruments for measuring violence and methods used to adjust for confounding factors). Some studies investigated only violence on the part of intimate partners and physical violence [1,2]. A review of African studies showed that most investigators elaborated their own questionnaires for the measurement of violence [2], impairing comparisons with other results. Many studies used logistic regression for data analysis [1,2], a statistical method that is not the most appropriate for testing associations related to complex phenomena such as violence [31,32].

In the present study, low social support, represented by the material, emotional/information, affective and positive social interaction dimensions, was associated with more episodes of general, psychological and physical/sexual violence. This negative effect was completely direct without being mediated by any of the variables analyzed. Thus, having better social support contributed to reduction of all types of violence [1,2,17,18].

In a study conducted in the municipality of Rio de Janeiro (Brazil), low social support assessed with the MOS instrument was associated with physical violence only in bivariate analysis [39]. A literature review pointed out that low levels of social support may be related to high levels of stress, thus contributing to an increased risk of violence [4].

The major strong point of the present study was the use of structural equation modeling in the analyses, which permitted us: a) to compare the factor structure of the measurement model of general, psychological and physical/sexual violence in two samples with contrasting socioeconomic conditions; b) to construct latent variables for the study of violence, social support and socioeconomic status, phenomena of difficult measurement; c) to improve the fit of the model initially proposed; and d) to assess the direct and indirect effect of socioeconomic status and social support on violence. A review of the literature did not identify studies using socioeconomic status, social support and violence as constructs or using structural equation modeling in their analyses [1–5,8]. A limitation of the present study was the fact that the subjects from both samples were part of a convenience sample that was not representative of the entire population of pregnant women in the municipalities. However, it is important to point out that nearly the same results of the final models were obtained for both samples, which gave consistency to the results.

This study did not address intimate partner violence but psychological, physical and sexual violence practiced by different persons in the family home environment and community, including also intimate partner violence. It is important to note that while most research have studied physical and/or sexual violence and we also investigated psychological violence, which is the most prevalent type. In addition, we analyzed separately those dimensions of violence (physical/sexual and psychological) as latent variables. Thus, this work differs from those already published in the literature because one of its goals was to determine if the effects of SES and social support on general violence (composed of physical, sexual and psychological dimensions) are different from those that are associated with its psychological and physical/sexual types. Another distinguishing feature of the study was that we evaluated SES and social support as latent variables, which reduces measurement error. The findings of this study suggest that strategies to prevent violence against pregnant women should include identification of women with low social support, a situation that put pregnant women at risk for all kinds of violence investigated.

## Supporting Information

S1 File Data from São Luís-Brazil, 2010–2011. (XLS)

S2 File Data from Ribeirão Preto-Brazil, 2010–2011. (XLS)
